# Supplementary material for: Ecological and geographical overlap drive plumage evolution and mimicry in woodpeckers
Source: Nat Commun. 2019 Apr 8;10:1602. doi: 10.1038/s41467-019-09721-w (PMC6453948; doi:10.1038/s41467-019-09721-w)
Supplement: Supplementary file 1 — Supplementary Info [file 41467_2019_9721_MOESM1_ESM.pdf]

Supplementary Information

Ecological and geographical overlap drive plumage evolution and mimicry in woodpeckers

Miller et al.

Table of Contents

Supplementary Tables 1-9
Supplementary Figures 1-18

Supplementary Table 1 | Principal components analysis of 230,000 CIELAB color values (1,000 samples from each of 230 woodpecker species). The first two PCs explain ~82% of the variance in color. PC1 has high loading values for luminance (L*) and yellowness (positive values of b*), and PC2 has high loading values for greenness (negative values of a*).

	PC1	PC2	PC3
Eigenvalues	1.17	1.04	0.74
Proportion of Variance	0.46	0.36	0.18
Cumulative Proportion of Variance	0.46	0.82	1
Color variable		*PC loadings*	
L*	**0.70**	0.29	-0.65
a*	0.01	**-0.92**	-0.39
b*	**0.71**	-0.27	0.65

Supplementary Table 2 | Principal components analysis of 138,000 CIELAB back color values (600 samples from each of 230 woodpecker species). The first two PCs explain ~85% of the variance in color. PC1 has high loading values for luminance (L*) and yellowness (positive values of b*), and PC2 has high loading values for greenness (negative values of a*).

	PC1	PC2	PC3
Eigenvalues	1.27	0.96	0.68
Proportion of Variance	0.54	0.31	0.15
Cumulative Proportion of Variance	0.54	0.85	1.00
Color variable		*PC loadings*	
L*	0.67	-0.21	**-0.71**
a*	-0.32	**-0.95**	-0.03
b*	0.67	-0.25	**0.70**

Supplementary Table 3 | Principal components analysis of 46,000 CIELAB head color values (200 samples from each of 230 woodpecker species). The first two PCs explain ~87% of the variance in color. PC1 has high loading values for greenness (negative values of a*) and blueness (negative values of b*), and PC2 has high loading values for luminance (L*).

	PC1	PC2	PC3
Eigenvalues	1.19	1.09	0.63
Proportion of Variance	0.47	0.39	0.13
Cumulative Proportion of Variance	0.47	0.87	1.00
Color variable		*PC loadings*	
L*	0.02	**0.88**	0.48
a*	**-0.71**	-0.33	0.62
b*	**-0.70**	0.35	-0.62

Supplementary Table 4 | Principal components analysis of 23,000 CIELAB belly color values (100 samples from each of 230 woodpecker species). The first two PCs explain ~81% of the variance in color. PC1 has high loading values for luminance (L*) and yellowness (positive values of b*), and PC2 has high loading values for greenness (negative values of a*).

	PC1	PC2	PC3
Eigenvalues	1.12	1.08	0.75
Proportion of Variance	0.42	0.39	0.19
Cumulative Proportion of Variance	0.42	0.81	1.00
Color variable		*PC loadings*	
L*	**0.70**	-0.39	0.60
a*	0.02	**0.85**	0.53
b*	**0.72**	0.35	-0.60

Supplementary Table 5 | Principal components analysis of pattern energy spectra across 230 species of woodpeckers. The three PC axes explain ~93% of the variance in pattern across all species.

	PC1	PC2	PC3
Standard deviation	3.08	2.12	1.34
Proportion of Variance	0.56	0.26	0.11
Cumulative Proportion	0.56	0.82	0.93

Supplementary Table 6 | Principal components analysis of pattern energy spectra across the backs of 230 species of woodpeckers. The three PC axes explain ~93% of the variance in pattern across all species.

	PC1	PC2	PC3
Standard deviation	2.85	2.46	1.29
Proportion of Variance	0.48	0.36	0.10
Cumulative Proportion	0.48	0.84	0.93

Supplementary Table 7 | Principal components analysis of pattern energy spectra across the heads of 230 species of woodpeckers. The three PC axes explain ~86% of the variance in pattern across all species.

	PC1	PC2	PC3
Standard deviation	2.80	1.91	1.75
Proportion of Variance	0.46	0.21	0.18
Cumulative Proportion	0.46	0.67	0.86

Supplementary Table 8 | Principal components analysis of pattern energy spectra across the bellies of 230 species of woodpeckers. The three PC axes explain ~92% of the variance in pattern across all species.

	PC1	PC2	PC3
Standard deviation	2.84	2.45	1.26
Proportion of Variance	0.48	0.35	0.09
Cumulative Proportion	0.48	0.83	0.92

Supplementary Table 9 | Specimens from the Cornell University Museum of Vertebrates (CUMV) used for color and pattern analyses.

Species (our taxonomy)	Species (on CUMV tag)	CUMV tag subspecies	CUMV collection number	Location
Xiphidiopicus percussus	*Xiphidiopicus percussus*	*percussus*	22746	Cuba
Xiphidiopicus percussus	*Xiphidiopicus percussus*	*percussus*	6498	Cuba
Xiphidiopicus percussus	*Xiphidiopicus percussus*	*percussus*	6497	Cuba
Sphyrapicus varius	*Sphyrapicus varius*	NA	55875	NY
Sphyrapicus varius	*Sphyrapicus varius*	NA	53166	NY
Sphyrapicus varius	*Sphyrapicus varius*	NA	30185	NY
Melanerpes carolinus	*Melanerpes carolinus*	NA	51400	NY
Melanerpes carolinus	*Melanerpes carolinus*	NA	55391	NY
Melanerpes carolinus	*Melanerpes carolinus*	NA	6198	TN
Dendrocopos major	*Dendrocopos major*	NA	6542	France
Dendrocopos major	*Dendrocopos major*	NA	6540	Germany
Dendrocopos major	*Dendrocopos major*	NA	6541	Italy
Colaptes rubiginosus	*Colaptes rubiginosus*	NA	34519	Chiapas
Colaptes rubiginosus	*Colaptes rubiginosus*	NA	6335	Motzorongo, Veracruz
Mesopicos goertae	*Mesopicos goertae*	NA	33636	Uganda
Mesopicos goertae	*Dendropicos spodocephalus*	NA	33640	Kenya
Mesopicos goertae	*Dendropicos spodocephalus*	*rhodeogaster*	6524	illegible
Picoides villosus	*Picoides villosus*	*villosus*	6406	NY
Picoides villosus	*Picoides villosus*	*villosus*	6407	NY
Picoides villosus	*Picoides villosus*	*villosus*	6415	NY
Picoides albolarvatus	*Picoides albolarvatus*	*albolarvatus*	6736	CA
Picoides albolarvatus	*Picoides albolarvatus*	*albolarvatus*	6733	CA
Picoides albolarvatus	*Picoides albolarvatus*	NA	22183	WA
Picus viridis	*Picus viridis*	*virescens*	7762	England
Picus viridis	*Picus viridis*	*virescens*	7763	England
Dendropicos fuscescens	*Dendropicos fuscescens*	*fuscescens*	6530	Angola
Dendropicos fuscescens	*Dendropicos fuscescens*	*fuscescens*	6529	Angola
Dendropicos fuscescens	*Dendropicos fuscescens*	NA	32433	Southwest Africa
Picoides pubescens	*Picoides pubescens*	*medianus*	6569	NY
Picoides pubescens	*Picoides pubescens*	*medianus*	6593	NY
Picoides pubescens	*Picoides pubescens*	*medianus*	6571	NY
Veniliornis kirkii	*Veniliornis kirkii*	NA	26606	Colombia
Veniliornis kirkii	*Veniliornis kirkii*	*neglectus*	6518	Costa Rica

Dryocopus pileatus	*Dryocopus pileatus*	NA	52879	NY
Dryocopus pileatus	*Dryocopus pileatus*	NA	54931	NY
Dryocopus pileatus	*Dryocopus pileatus*	NA	53082	NY
Dinopium benghalense	*Dinopium benghalense*	*benghalense*	23454	India
Campephilus guatemalensis	*Campephilus guatemalensis*	NA	29193	Honduras
Campephilus guatemalensis	*Campephilus guatemalensis*	*guatemalensis*	6397	Costa Rica
Campephilus guatemalensis	*Campephilus guatemalensis*	*regius*	6402	Mexico
Campethera nivosa	*Campethera nivosa*	*efulenensis*	6519	Cameroon
Campethera nivosa	*Campethera nivosa*	NA	29775	Nigeria
Campethera nivosa	*Campethera nivosa*	NA	33632	Uganda
Gecinulus grantia	*Gecinulus grantia*	NA	6464	India
Blythipicus pyrrhotis	*Blythipicus pyrrhotis*	NA	6479	India
Hemicircus canente	*Hemicircus canente*	*canente*	6501	India
Chrysocolaptes guttacristatus	*Chrysocolaptes guttacristatus*	NA	6486	illegible
Chrysocolaptes guttacristatus	*Chrysocolaptes guttacristatus*	NA	23486	Burma
Colaptes auratus	*Colaptes auratus*	NA	48699	NY
Colaptes auratus	*Colaptes auratus*	NA	28168	NY
Colaptes auratus	*Colaptes auratus*	NA	14975	NY
Colaptes auratus	*Colaptes auratus*	NA	48717	NY
Meiglyptes tristis	*Meiglyptes tristis*	*grammithorax*	6466	India
Celeus castaneus	*Celeus castaneus*	NA	6471	Mexico
Celeus castaneus	*Celeus castaneus*	NA	6473	Costa Rica
Celeus castaneus	*Celeus castaneus*	NA	6472	Costa Rica

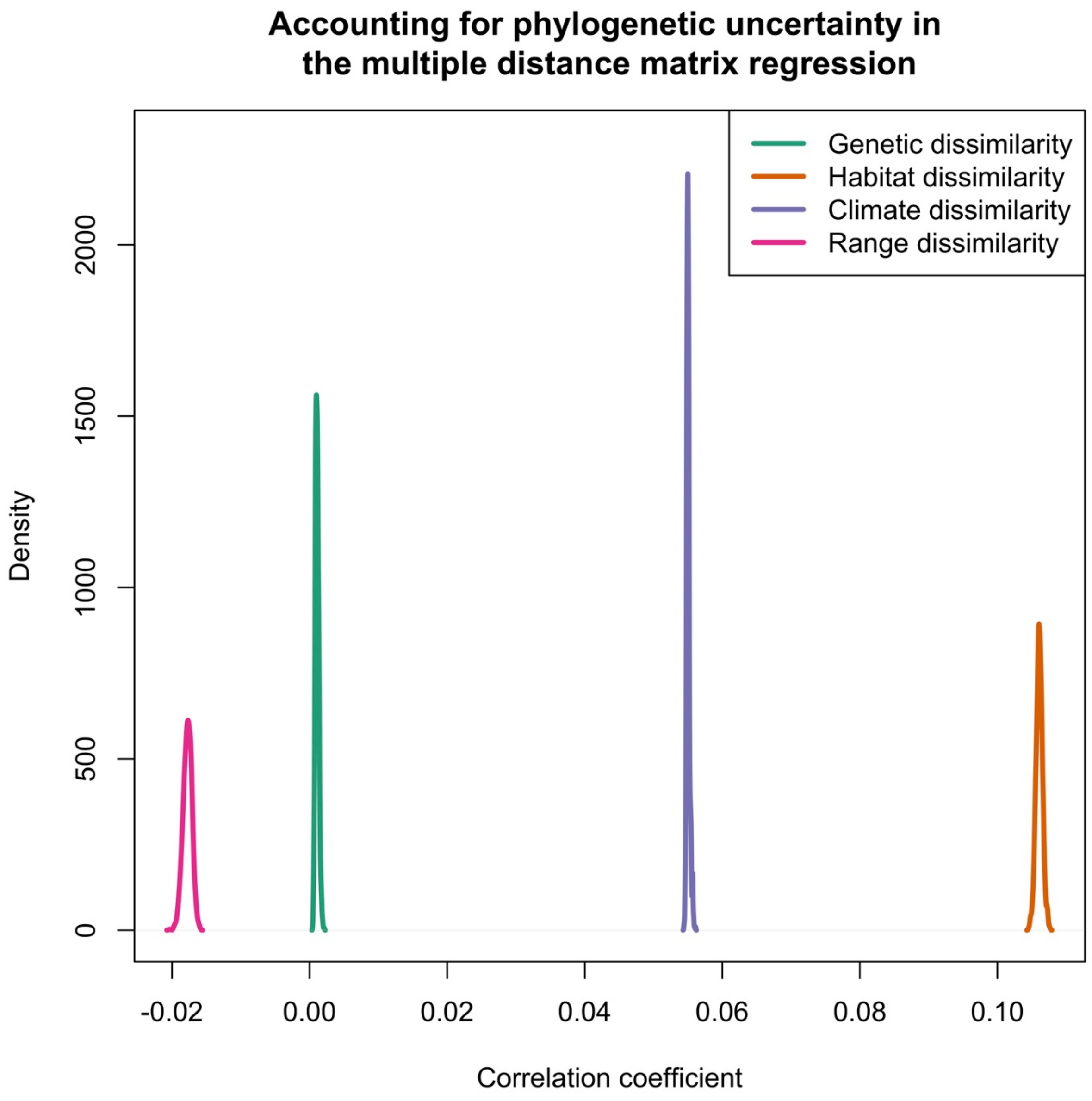

Supplementary Fig. 1 | Correlations between plumage and each of the independent variables are not strongly influenced by phylogenetic uncertainty. Correlation coefficients between the independent matrices (genetic, habitat, and climate dissimilarity) and the dependent matrix (plumage dissimilarity) have narrow distributions (kernel density estimates shown).

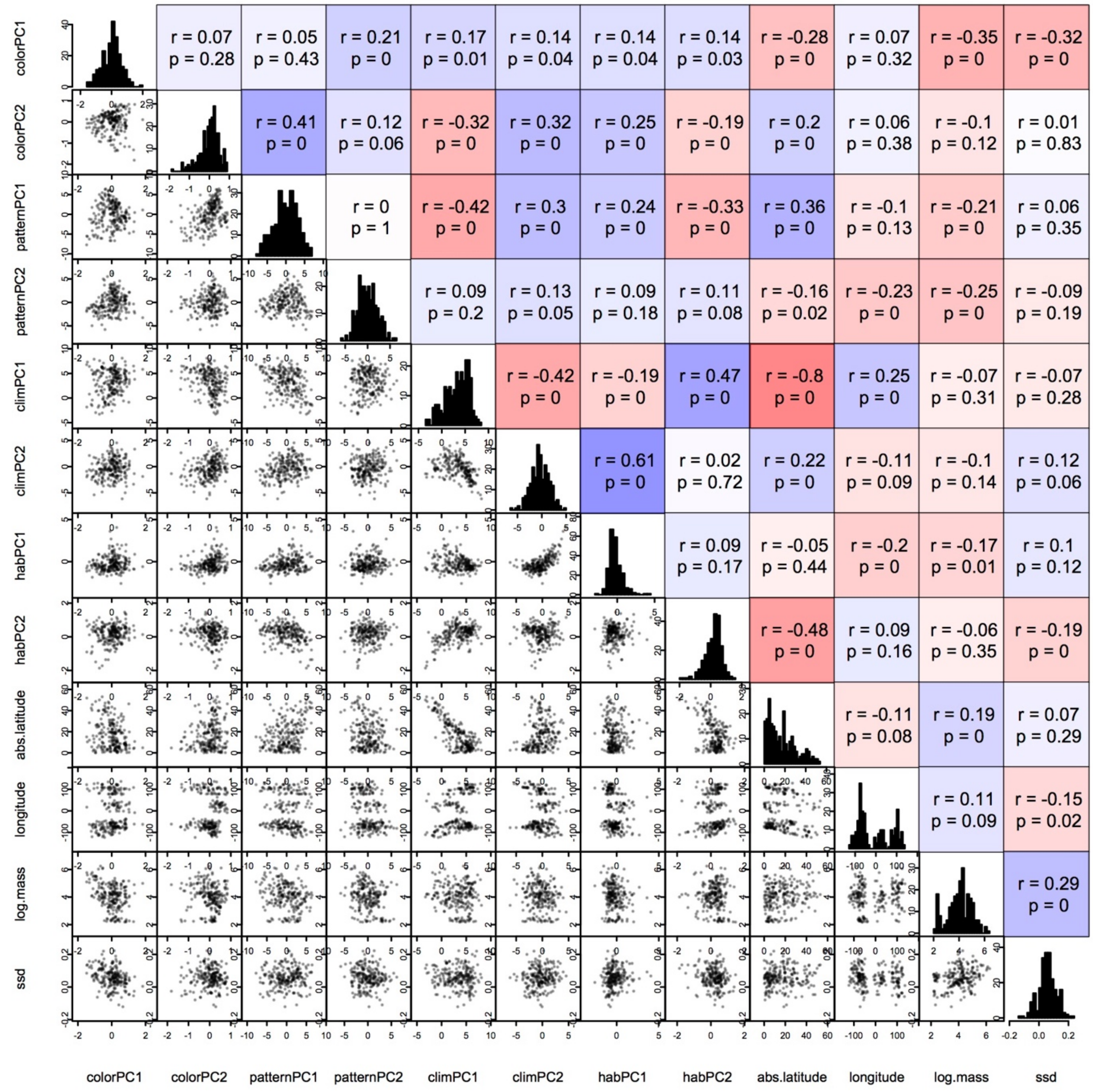

Supplementary Fig. 2 | Paneled correlation plot showing the relationships between all species-level averaged traits. Climate, habitat, latitude, and woodpecker body mass and plumage appearance exhibit a variety of intercorrelations. The lower triangle panels show the pairwise trait relationships. The diagonal panels contain histograms of the indicated variable. The upper triangle panels summarize the Pearson's correlation coefficient and the significance of the relationship, both numerically and in color, where bright red indicates a strong negative correlation, and bright blue indicates a strong positive correlation.

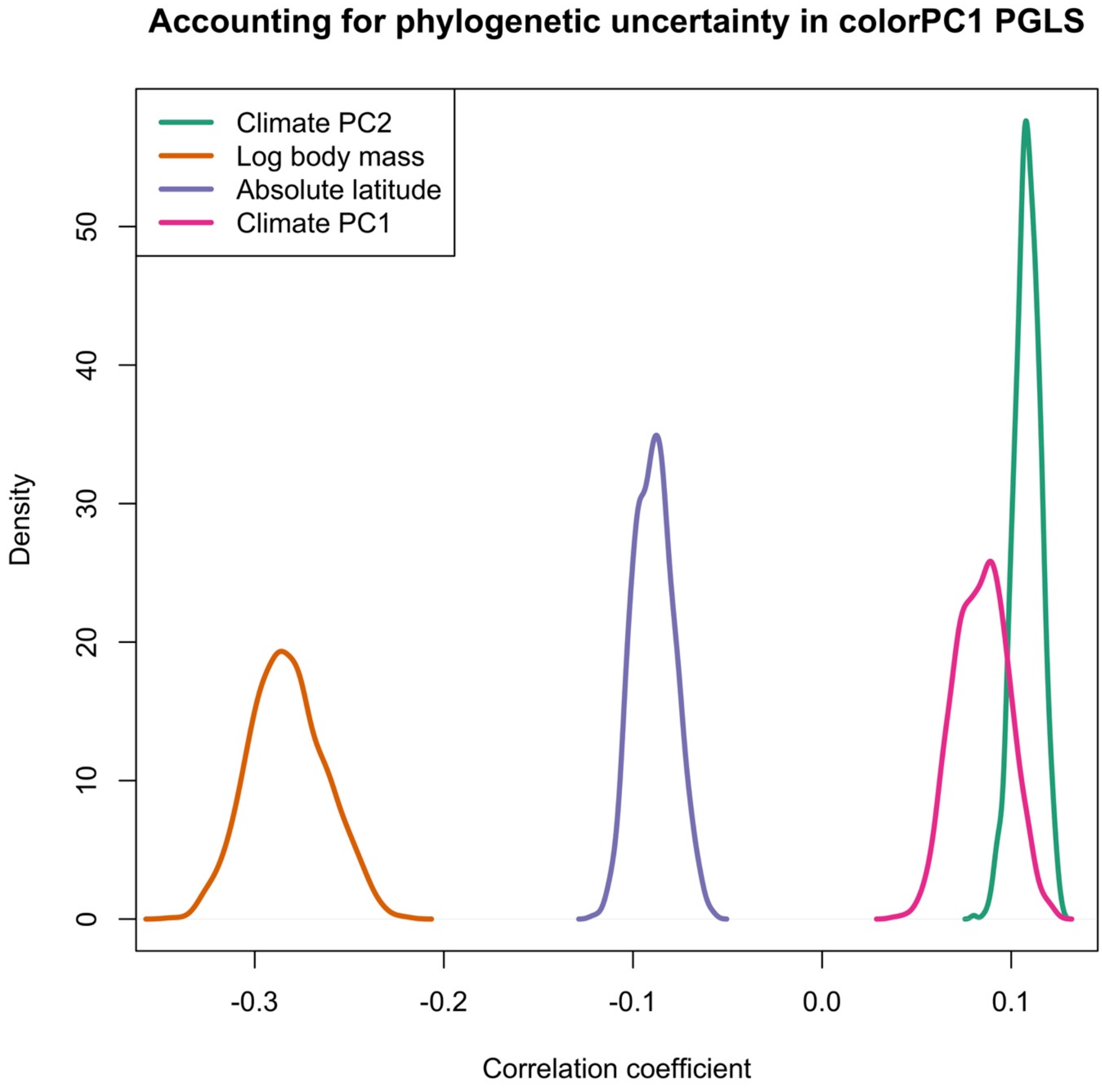

Supplementary Fig. 3 | Correlations between colorPC1 and each of the independent variables are not strongly influenced by phylogenetic uncertainty. Correlation coefficients between the independent variables and colorPC1 have narrow distributions (kernel density estimates shown).

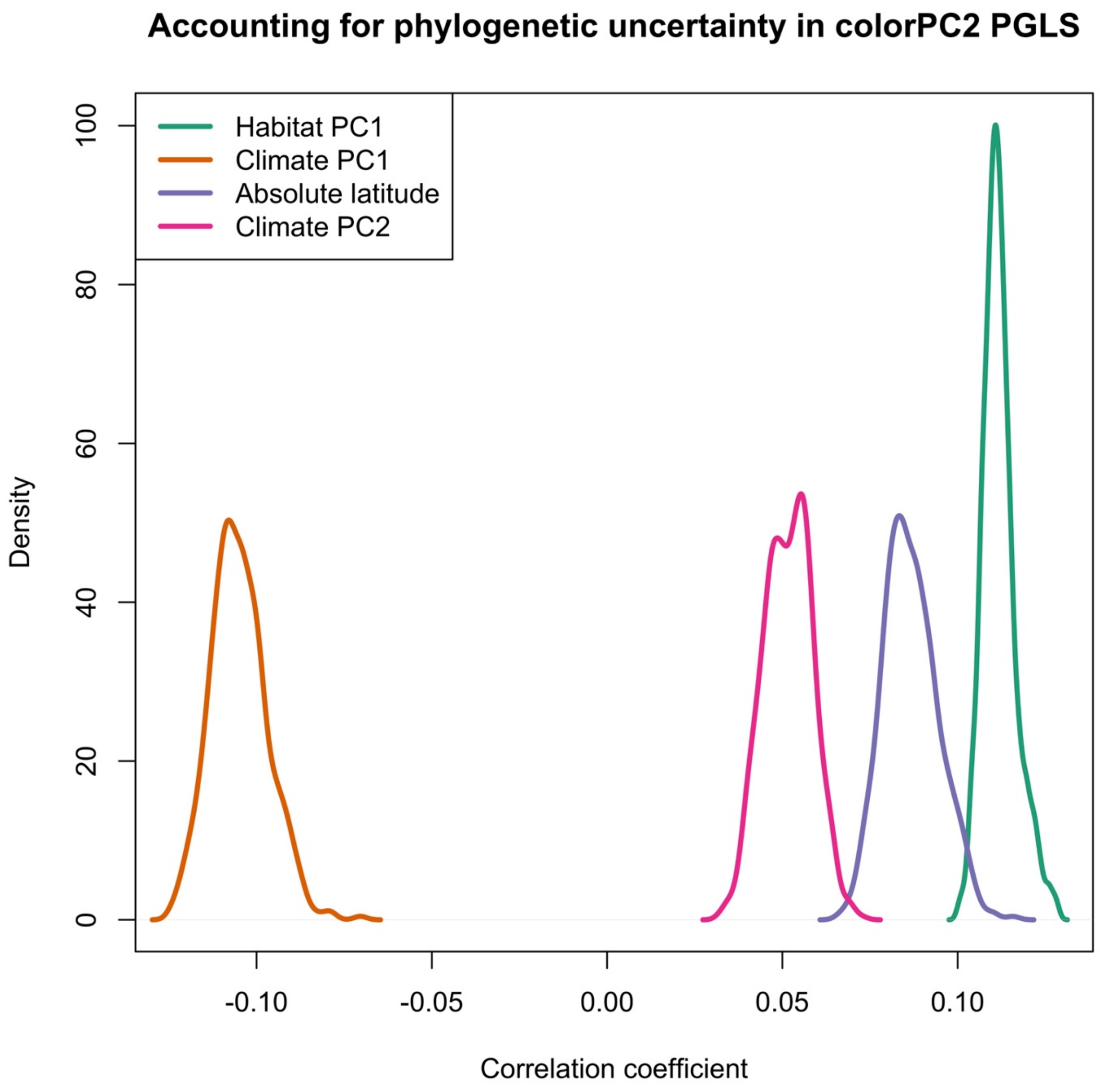

Supplementary Fig. 4 | Correlations between colorPC1 and each of the independent variables are not strongly influenced by phylogenetic uncertainty. Correlation coefficients between the independent variables and colorPC2 have narrow distributions (kernel density estimates shown).

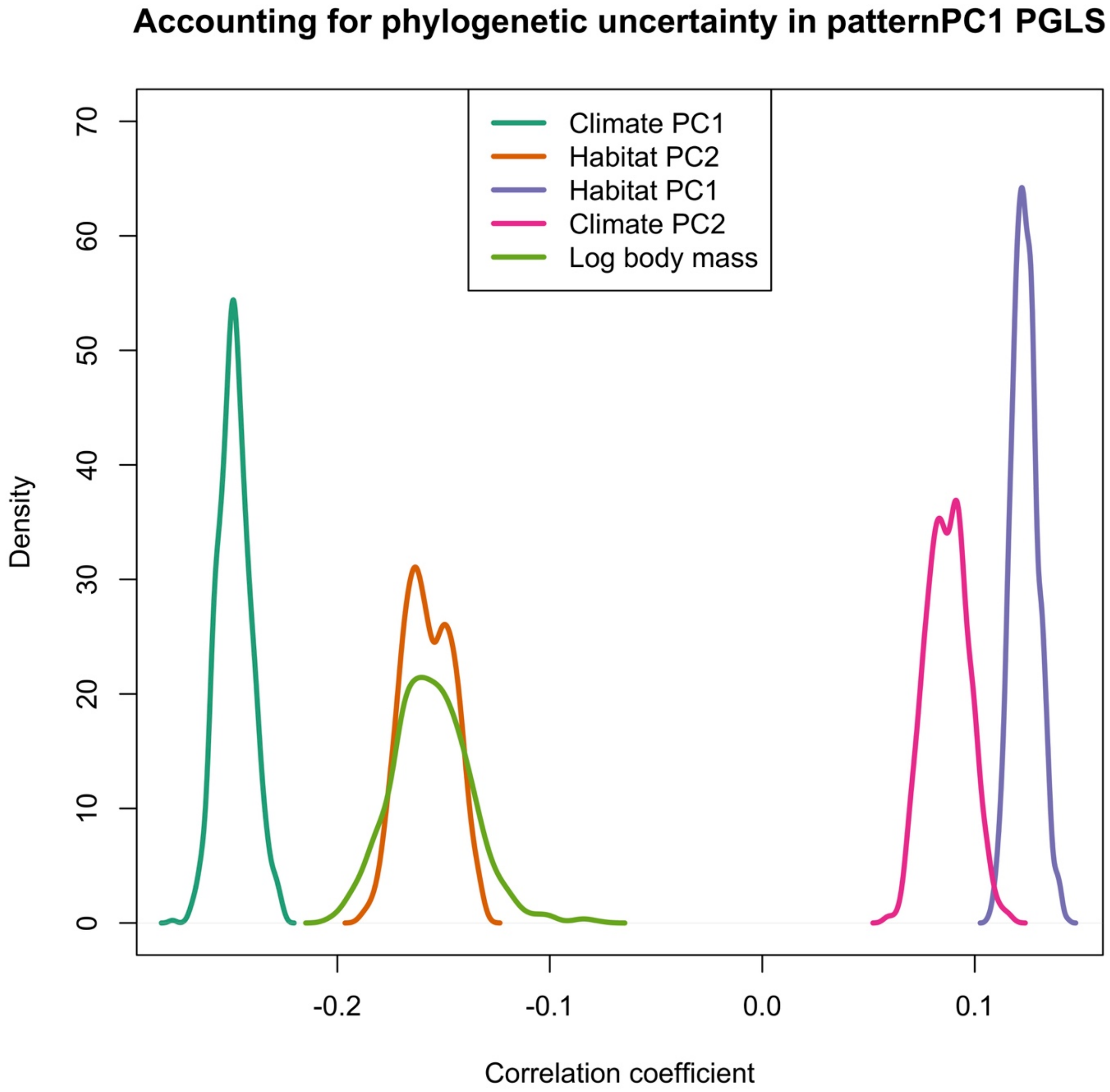

Supplementary Fig. 5 | Correlations between colorPC1 and each of the independent variables are not strongly influenced by phylogenetic uncertainty. Correlation coefficients between the independent variables and patternPC1 have narrow distributions (kernel density estimates shown).

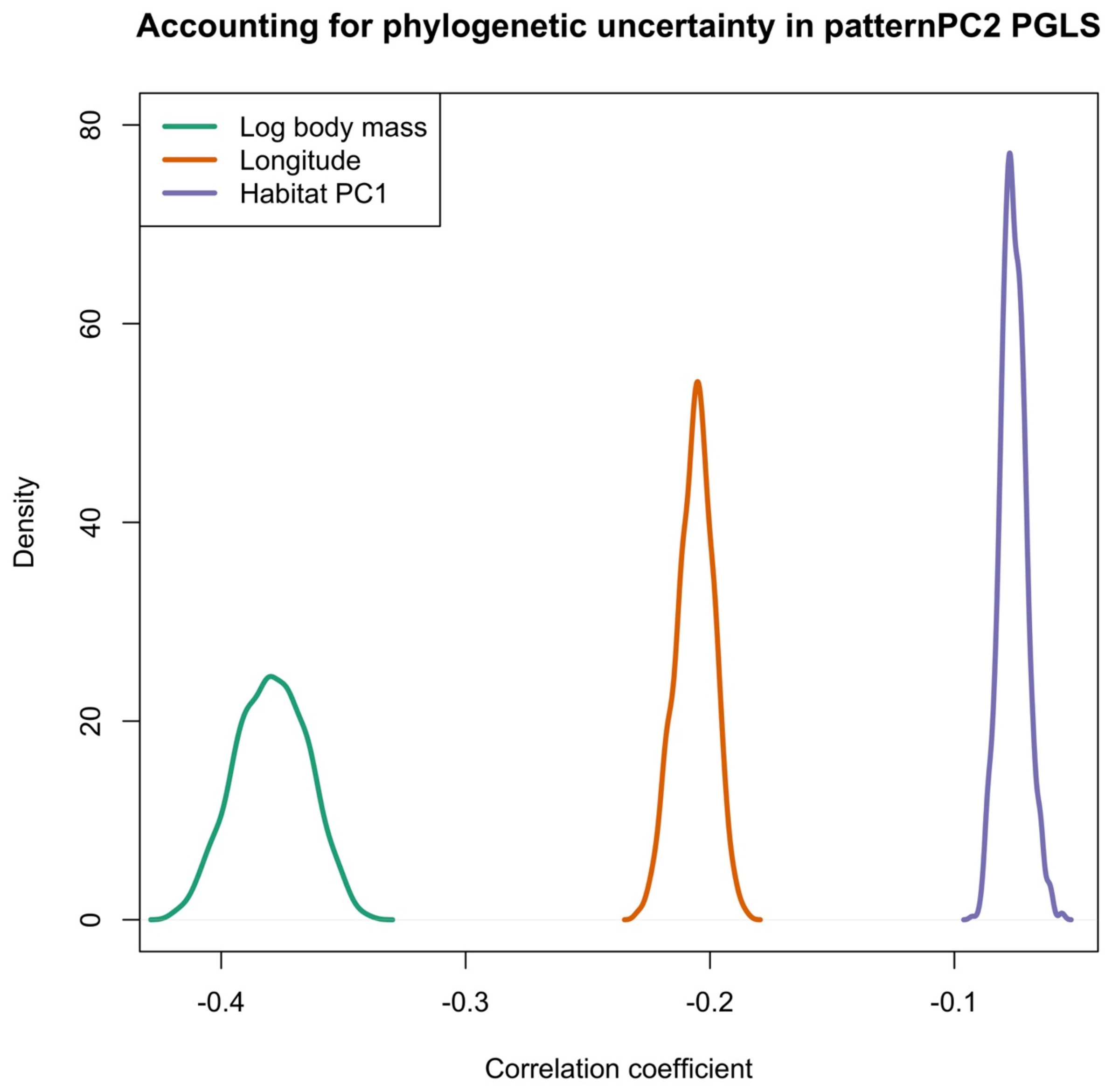

Supplementary Fig. 6 | Correlations between colorPC1 and each of the independent variables are not strongly influenced by phylogenetic uncertainty. Correlation coefficients between the independent variables and patternPC2 have narrow distributions (kernel density estimates shown).

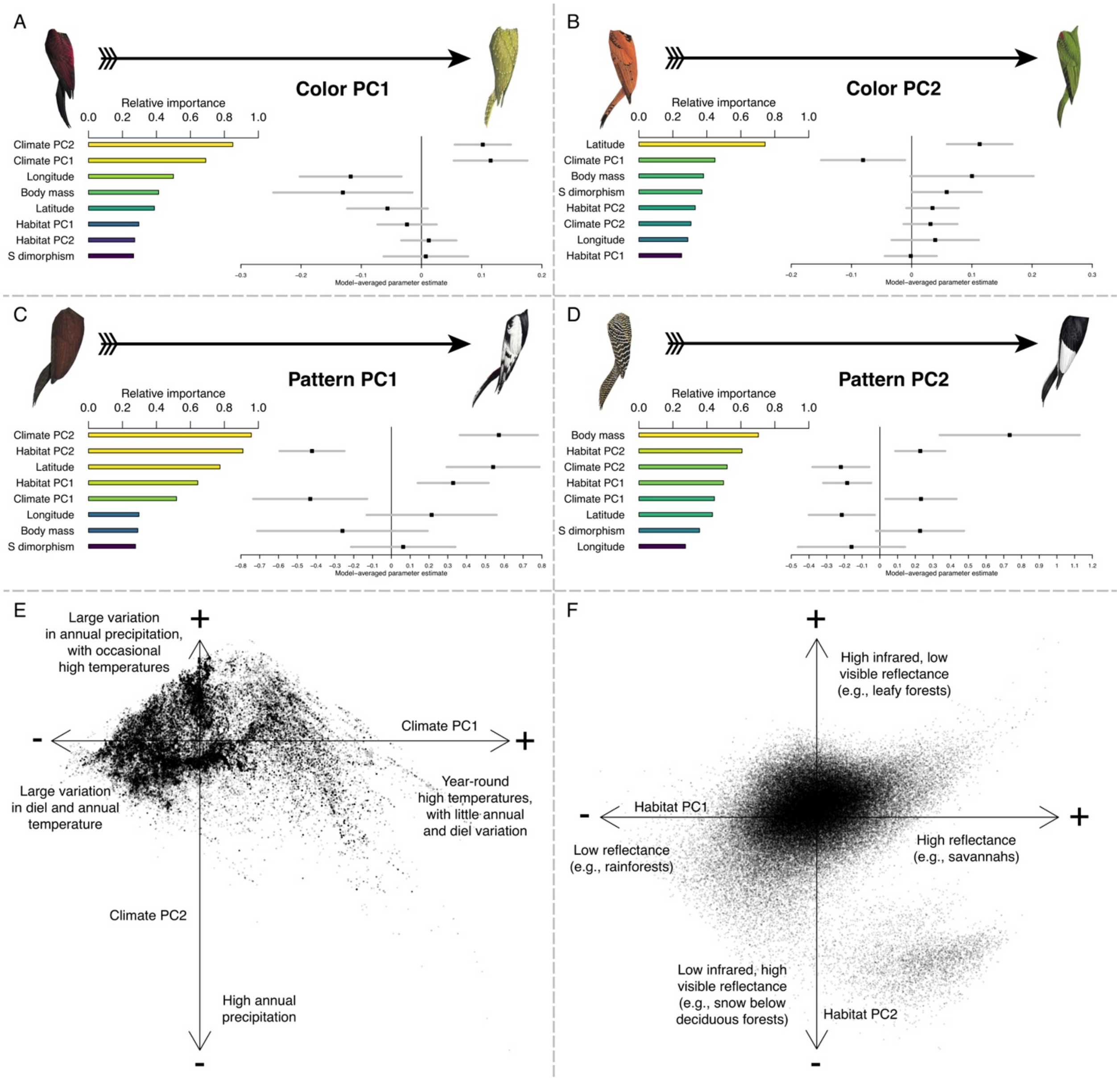

Supplementary Fig. 7 | Back-specific variable importance scores and model-averaged parameter estimates from phylogenetic generalized least squares regressions. These quantify how colour and pattern vary as a function of climate, habitat, body mass, sexual size dimorphism, latitude and longitude, with summaries of the climate and habitat principal component analyses (PCA). Model-averaged *p*-values of explanatory factors are colour-coded from yellow to blue; only factors with *p*-values < 0.05 are coloured yellow and discussed here. (a) Dark birds are heavier and occur in wetter climates. (b) Greenish (as opposed to reddish) birds are found in more open habitats. (c) Less-patterned birds are found in aseasonal climates, open habitats, and temperate forests. (d) Birds patterned in large plumage elements, such as large colour patches, tend to be larger in body size. (e) Climate PCA results, illustrating the distribution of woodpeckers in climate space, with qualitative descriptions of the first two PC axes. (f) Habitat PCA results, showing the distribution of woodpeckers across global habitats, with qualitative descriptions of the first two PC axes. Illustrations © HBW Alive/Lynx Edicions.

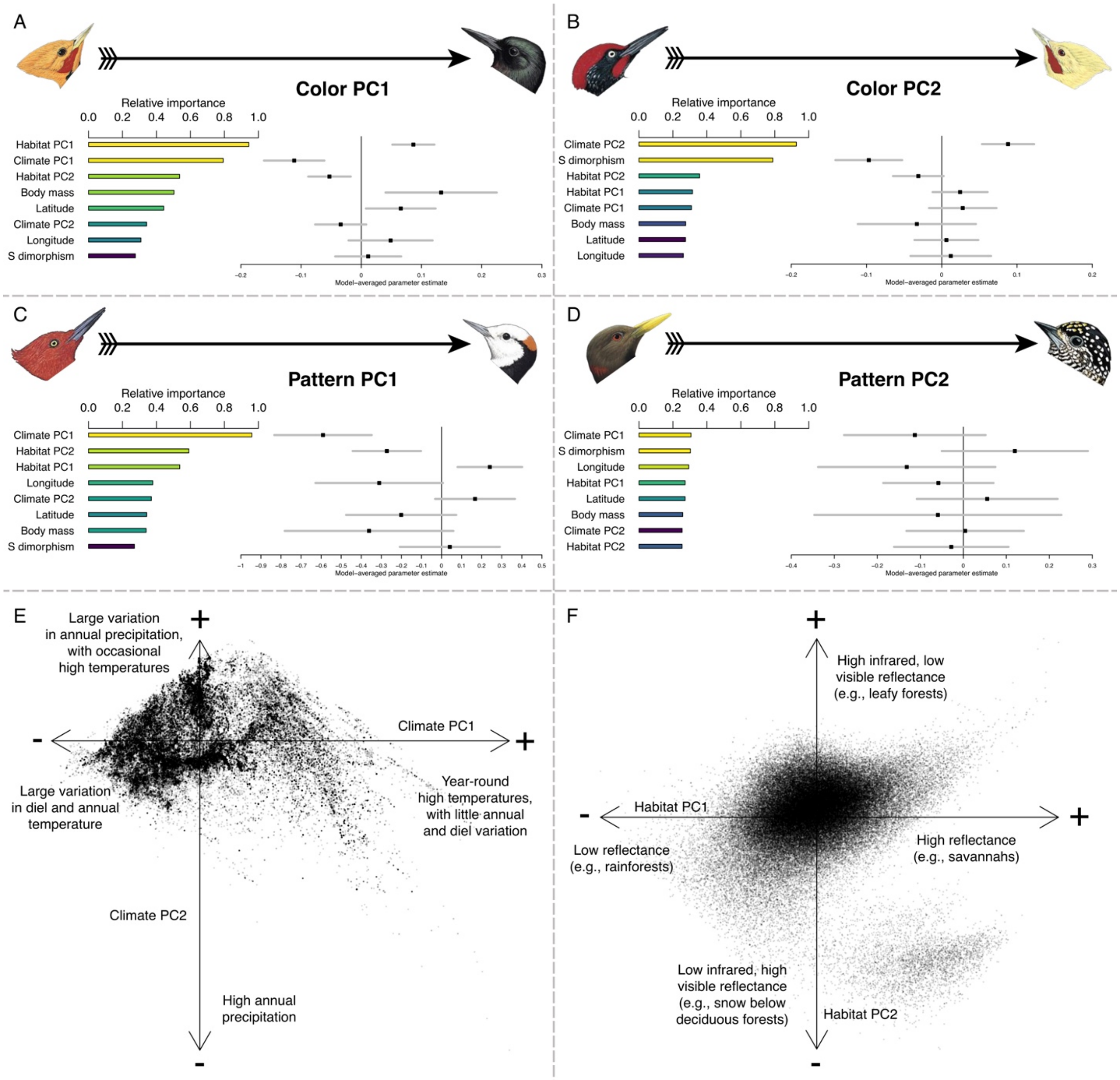

Supplementary Fig. 8 | Head-specific variable importance scores and model-averaged parameter estimates from phylogenetic generalized least squares regressions. These quantify how colour and pattern vary as a function of climate, habitat, body mass, sexual size dimorphism, latitude and longitude, with summaries of the climate and habitat principal component analyses (PCA). Model-averaged *p*-values of explanatory factors are colour-coded from yellow to blue; only factors with *p*-values < 0.05 are coloured yellow and discussed here. (a) Dark birds are heavier and occur in wetter climates. (b) Greenish (as opposed to reddish) birds are found in more open habitats. (c) Less-patterned birds are found in aseasonal climates, open habitats, and temperate forests. (d) Birds patterned in large plumage elements, such as large colour patches, tend to be larger in body size. (e) Climate PCA results, illustrating the distribution of woodpeckers in climate space, with qualitative descriptions of the first two PC axes. (f) Habitat PCA results, showing the distribution of woodpeckers across global habitats, with qualitative descriptions of the first two PC axes. Illustrations © HBW Alive/Lynx Edicions.

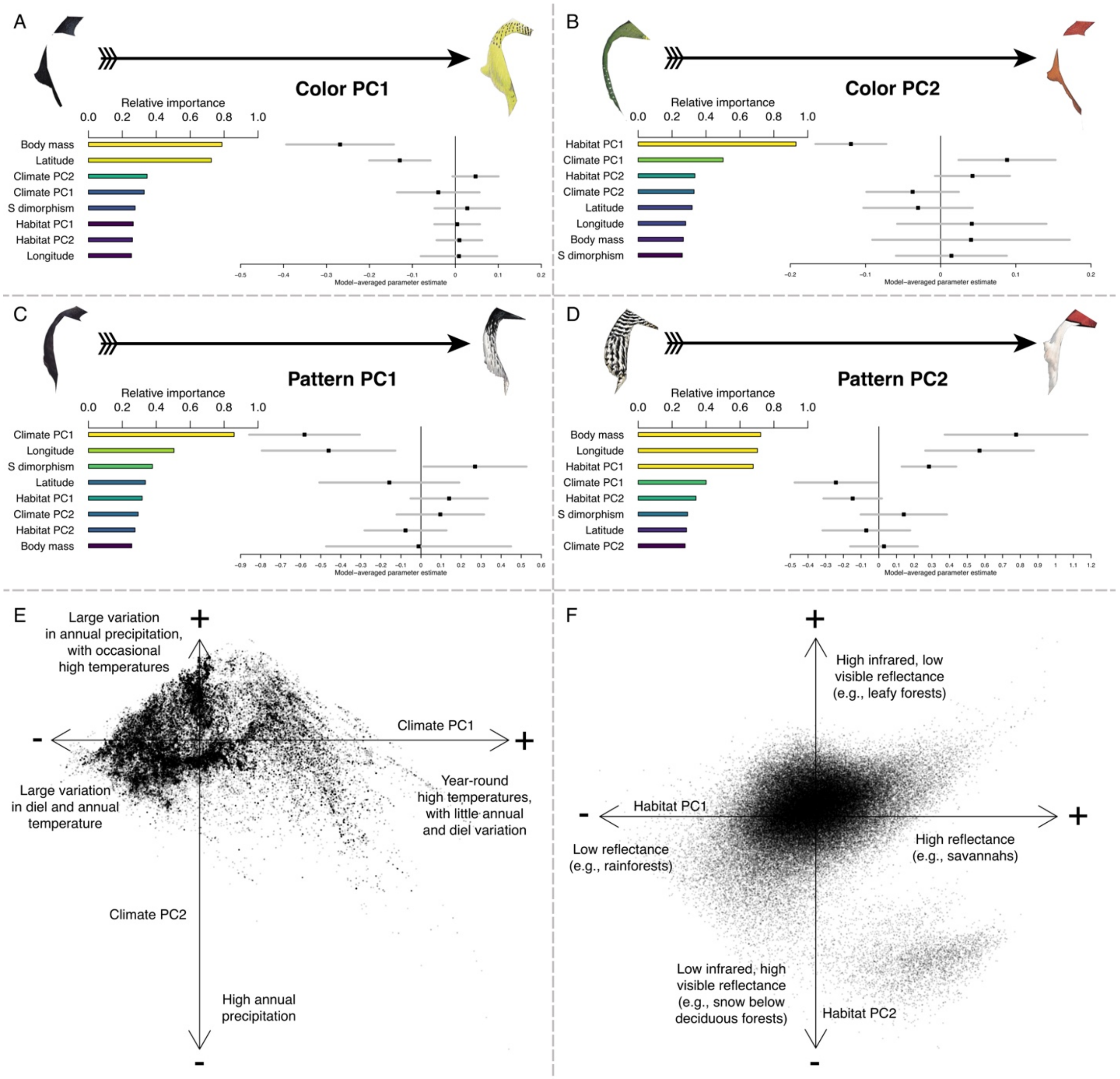

Supplementary Fig. 9 | Belly-specific variable importance scores and model-averaged parameter estimates from phylogenetic generalized least squares regressions. These quantify how colour and pattern vary as a function of climate, habitat, body mass, sexual size dimorphism, latitude and longitude, with summaries of the climate and habitat principal component analyses (PCA). Model-averaged *p*-values of explanatory factors are colour-coded from yellow to blue; only factors with *p*-values < 0.05 are coloured yellow and discussed here. (a) Dark birds are heavier and occur in wetter climates. (b) Greenish (as opposed to reddish) birds are found in more open habitats. (c) Less-patterned birds are found in aseasonal climates, open habitats, and temperate forests. (d) Birds patterned in large plumage elements, such as large colour patches, tend to be larger in body size. (e) Climate PCA results, illustrating the distribution of woodpeckers in climate space, with qualitative descriptions of the first two PC axes. (f) Habitat PCA results, showing the distribution of woodpeckers across global habitats, with qualitative descriptions of the first two PC axes. Illustrations © HBW Alive/Lynx Edicions.

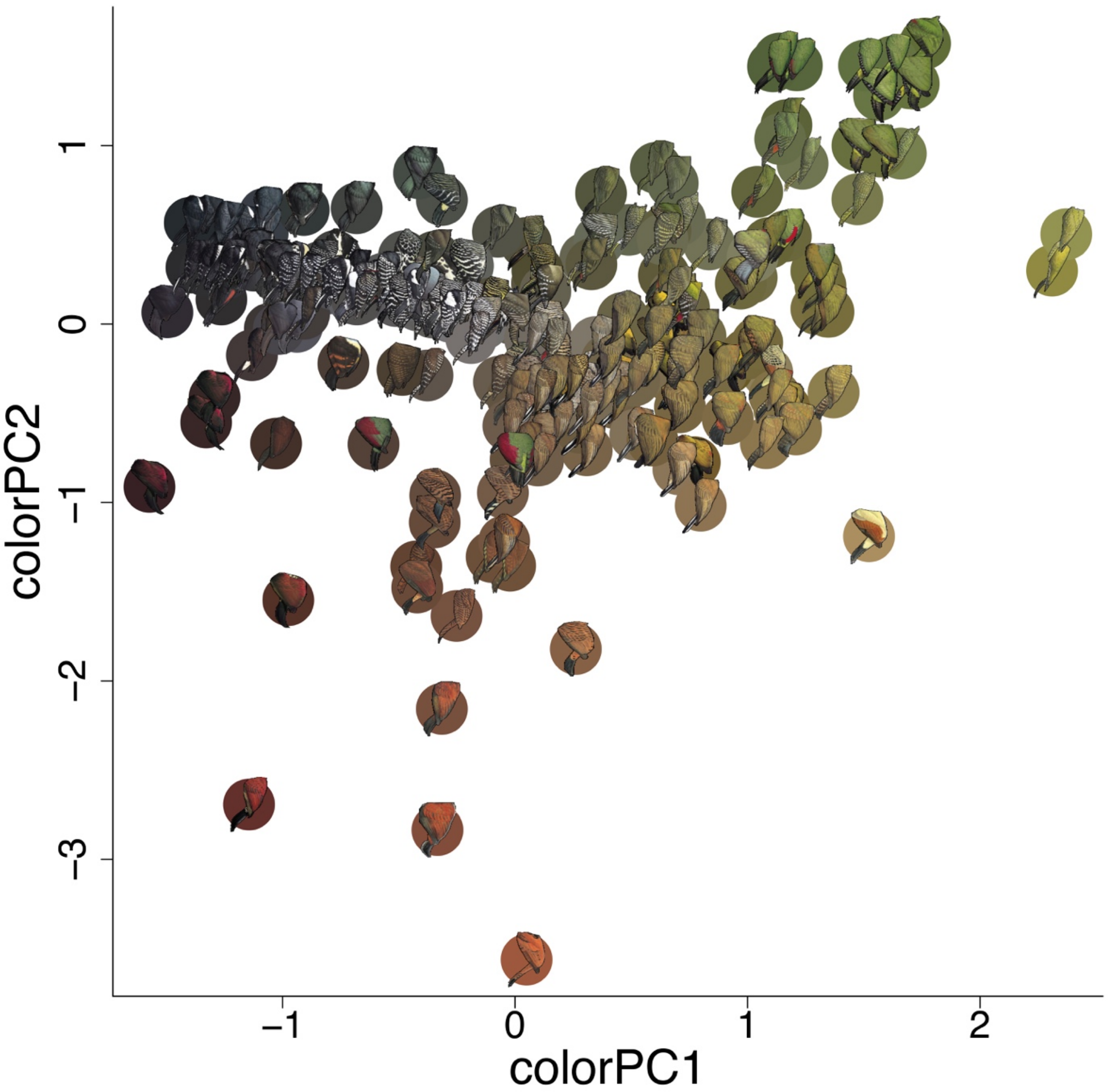

Supplementary Fig. 10 | Back-specific principal components analysis (PCA) of species-averaged woodpecker colour values. Principal component one (colourPC1) explains 45% of the variation in measured colour scores. Higher PC1 scores correspond to greater luminance values, and more yellow and less blue. Principal component two (colourPC2) explains an additional 36% of variation in overall colour scores. Higher PC2 scores correspond to more green and less red colouration. Coloured circles behind each woodpecker species correspond to the average CIE L*a*b scores for the 600 randomly selected colour samples from that species' back. Illustrations © HBW Alive/Lynx Edicions.

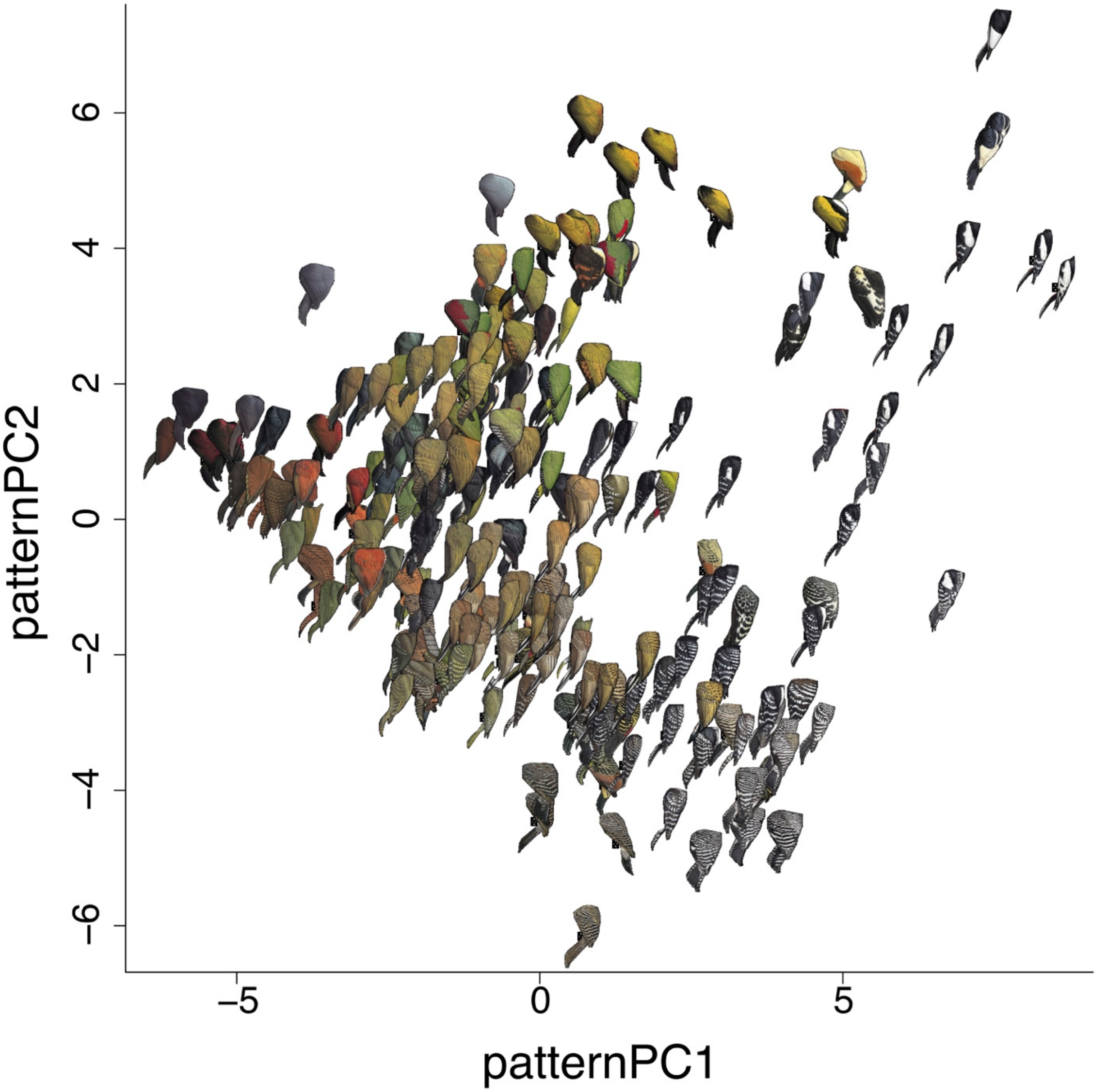

Supplementary Fig. 11 | Back-specific major axes of plumage pattern variation quantified using granularity analysis, then summarized with a principal components analysis (PCA) for species-level approaches. Pattern PC1 and PC2, collectively, account for 82.1% of variation across woodpeckers. Exemplar species (i-iv) illustrate extreme variation in PC1: (i) exhibits high energy scores across most pattern element sizes, with small, medium, and large pattern elements; (ii) has low energy scores across the spectrum, with few pattern elements of any size. And, extreme variation in PC2: (iii) has many small pattern elements and few of any other sizes; (iv) has only medium and large size pattern elements. Illustrations © HBW Alive/Lynx Edicions.

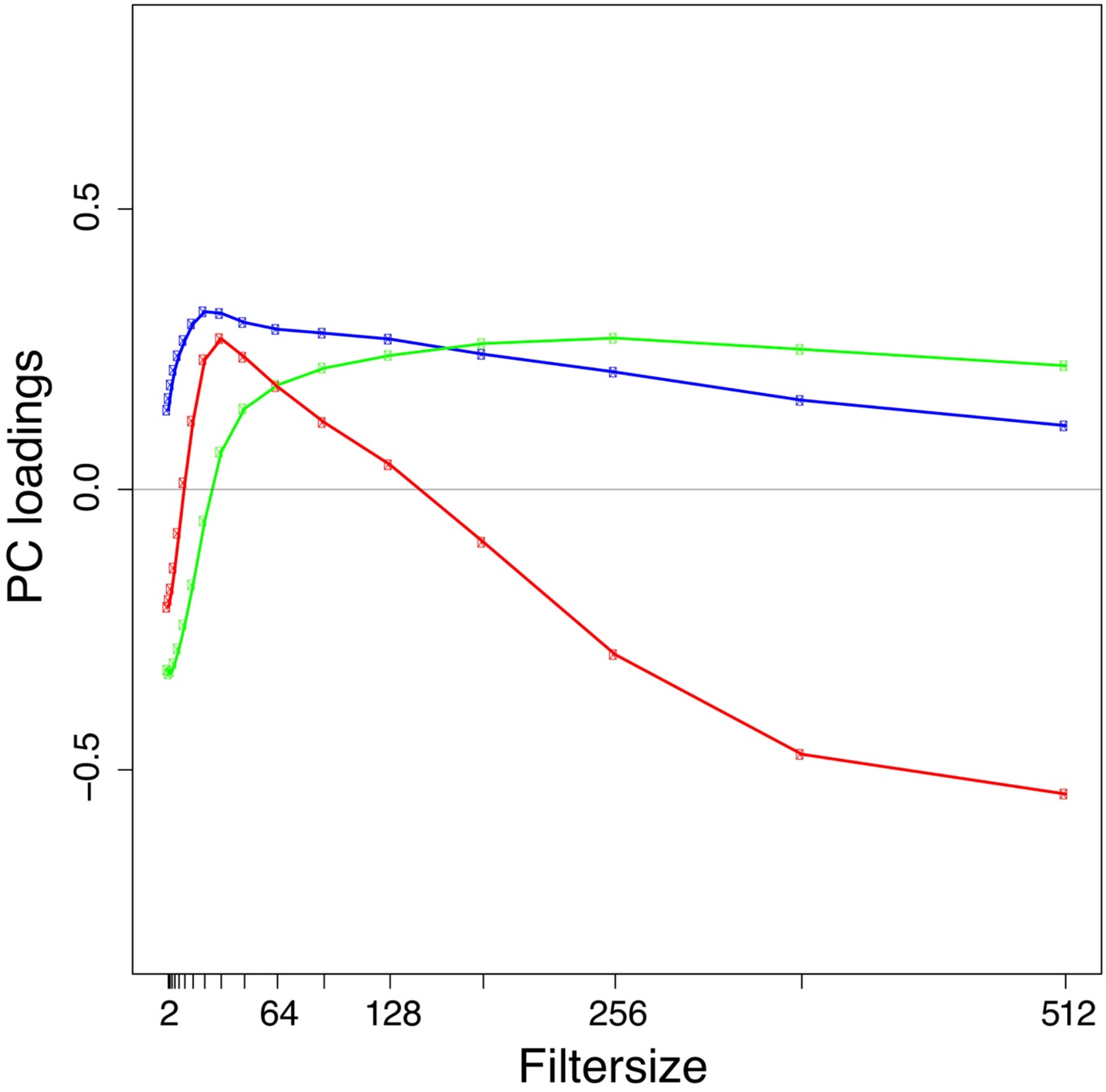

Supplementary Fig. 12 | Principal component loadings of back pattern. Principal component loadings of pattern energy spectra across filter size for all species reveals how back pattern elements of different sizes influence PC scores.

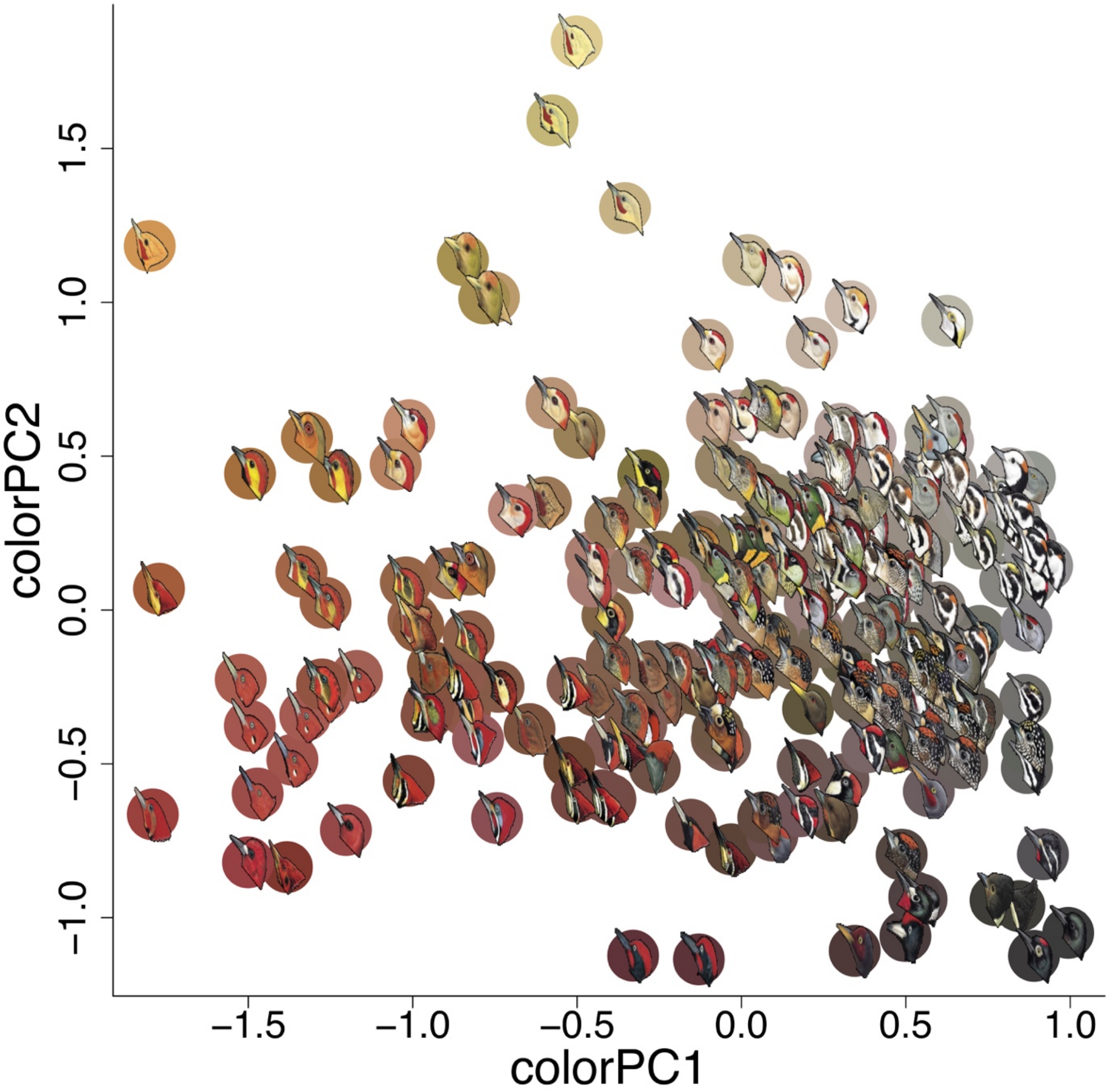

Supplementary Fig. 13 | Head-specific principal components analysis (PCA) of species-averaged woodpecker colour values. Principal component one (colourPC1) explains 45% of the variation in measured colour scores. Higher PC1 scores correspond to greater luminance values, and more yellow and less blue. Principal component two (colourPC2) explains an additional 36% of variation in overall colour scores. Higher PC2 scores correspond to more green and less red colouration. Coloured circles behind each woodpecker species correspond to the average CIE L*a*b scores for the 200 randomly selected colour samples from that species' head. Illustrations © HBW Alive/Lynx Edicions.

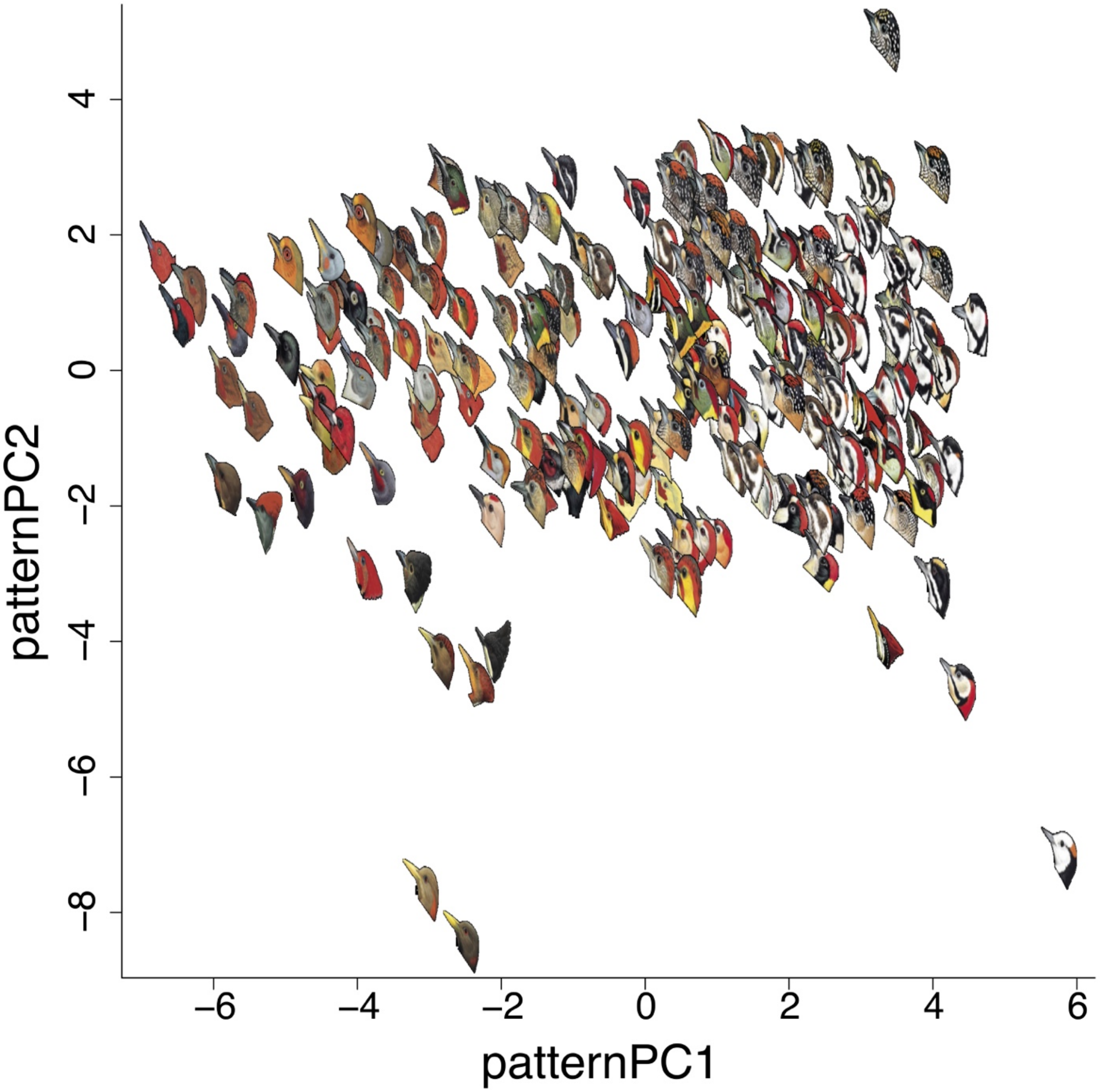

Supplementary Fig. 14 | Head-specific major axes of plumage pattern variation quantified using granularity analysis, then summarized with a principal components analysis (PCA) for species-level approaches. Pattern PC1 and PC2, collectively, account for 82.1% of variation across woodpeckers. Exemplar species (i-iv) illustrate extreme variation in PC1: (i) exhibits high energy scores across most pattern element sizes, with small, medium, and large pattern elements; (ii) has low energy scores across the spectrum, with few pattern elements of any size. And, extreme variation in PC2: (iii) has many small pattern elements and few of any other sizes; (iv) has only medium and large size pattern elements. Illustrations © HBW Alive/Lynx Edicions.

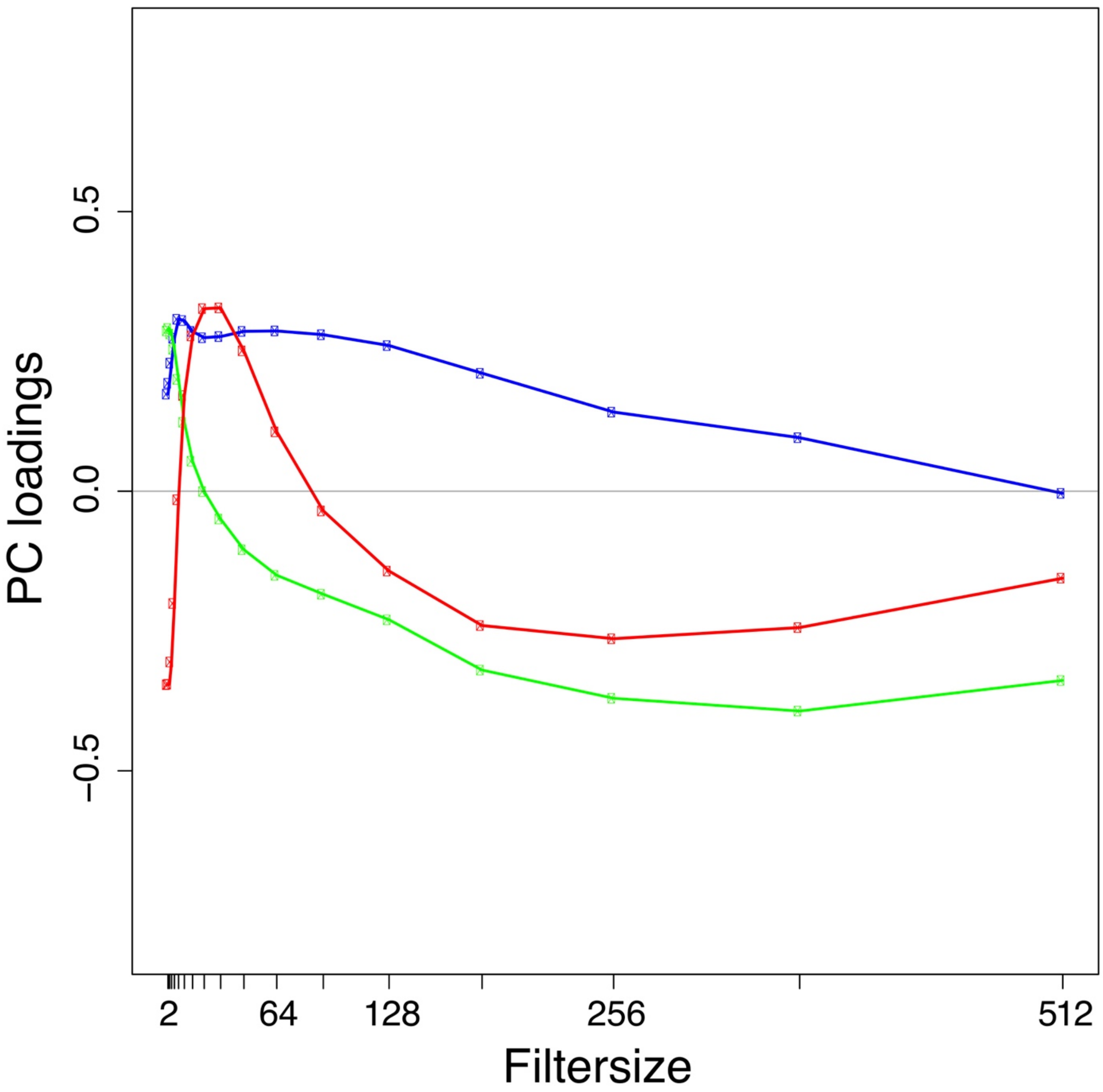

Supplementary Fig. 15 | Principal component loadings of head pattern. Principal component loadings of pattern energy spectra across filter size for all species reveals how head pattern elements of different sizes influence PC scores.

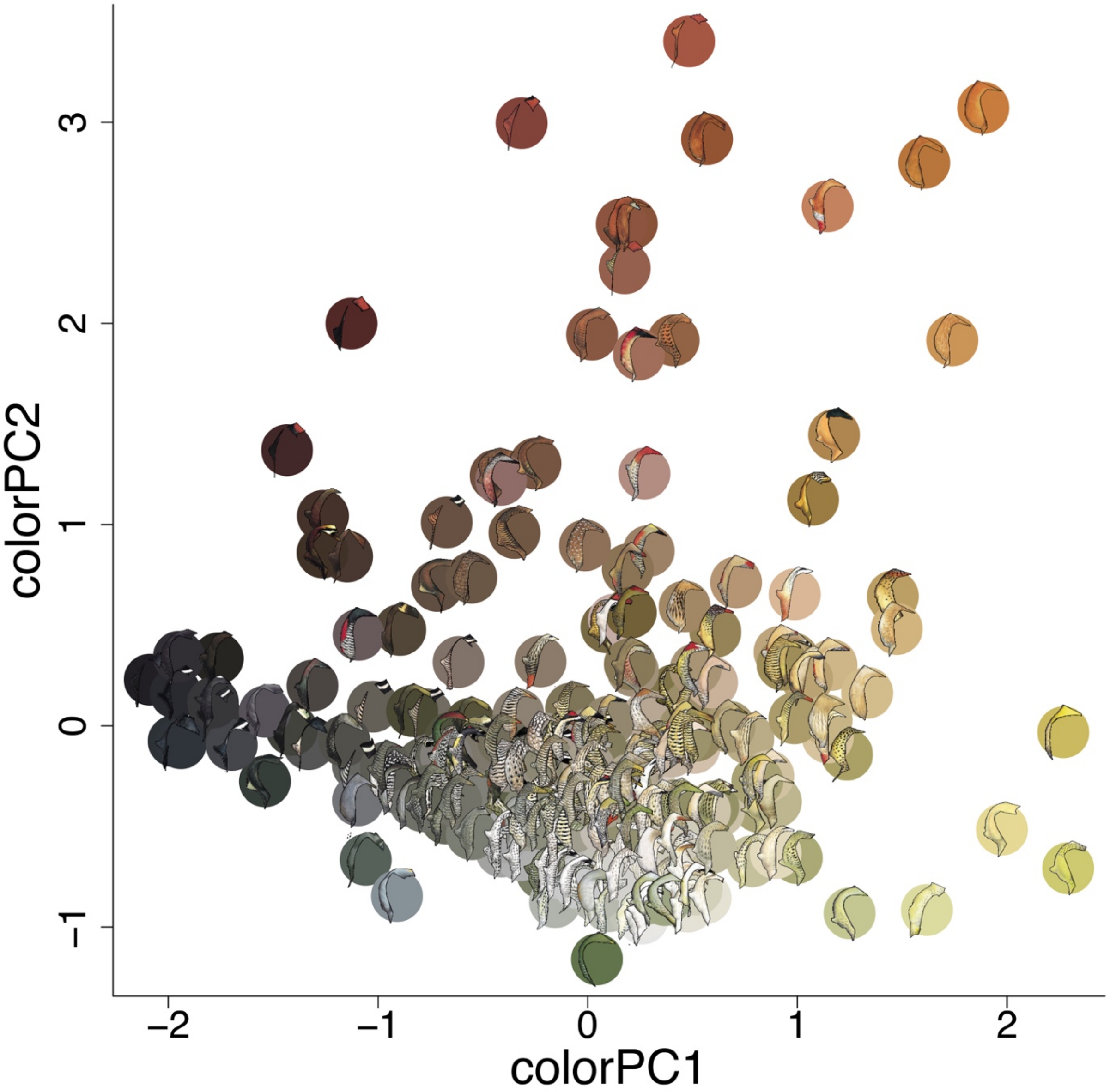

Supplementary Fig. 16 | Belly-specific principal components analysis (PCA) of species-averaged woodpecker colour values. Principal component one (colourPC1) explains 45% of the variation in measured colour scores. Higher PC1 scores correspond to greater luminance values, and more yellow and less blue. Principal component two (colourPC2) explains an additional 36% of variation in overall colour scores. Higher PC2 scores correspond to more green and less red colouration. Coloured circles behind each woodpecker species correspond to the average CIE L*a*b scores for the 100 randomly selected colour samples from that species' belly. Illustrations © HBW Alive/Lynx Edicions.

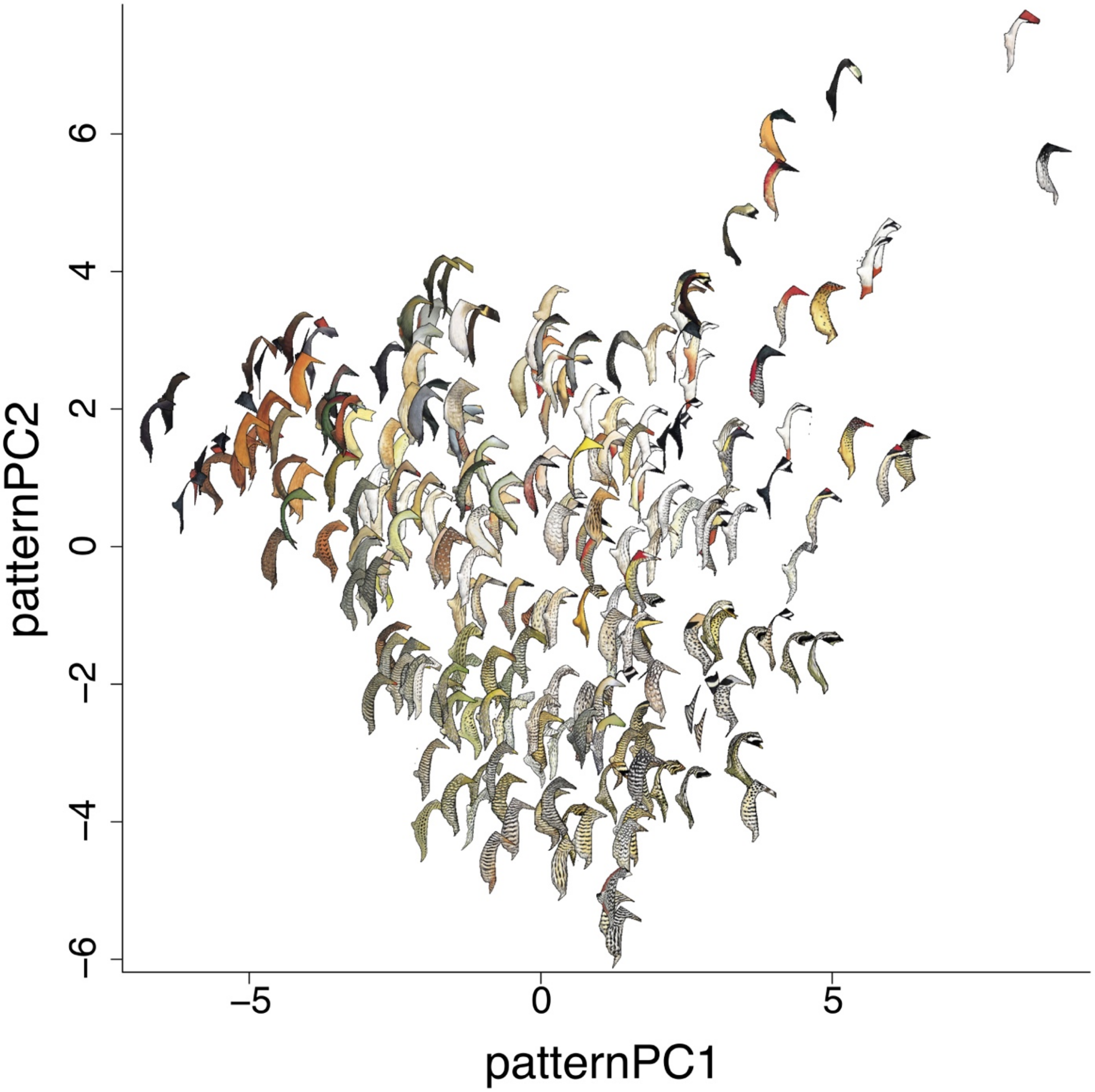

Supplementary Fig. 17 | Belly-specific major axes of plumage pattern variation quantified using granularity analysis, then summarized with a principal components analysis (PCA) for species-level approaches. Pattern PC1 and PC2, collectively, account for 82.1% of variation across woodpeckers. Exemplar species (i-iv) illustrate extreme variation in PC1: (i) exhibits high energy scores across most pattern element sizes, with small, medium, and large pattern elements; (ii) has low energy scores across the spectrum, with few pattern elements of any size. And, extreme variation in PC2: (iii) has many small pattern elements and few of any other sizes; (iv) has only medium and large size pattern elements. Illustrations © HBW Alive/Lynx Edicions.

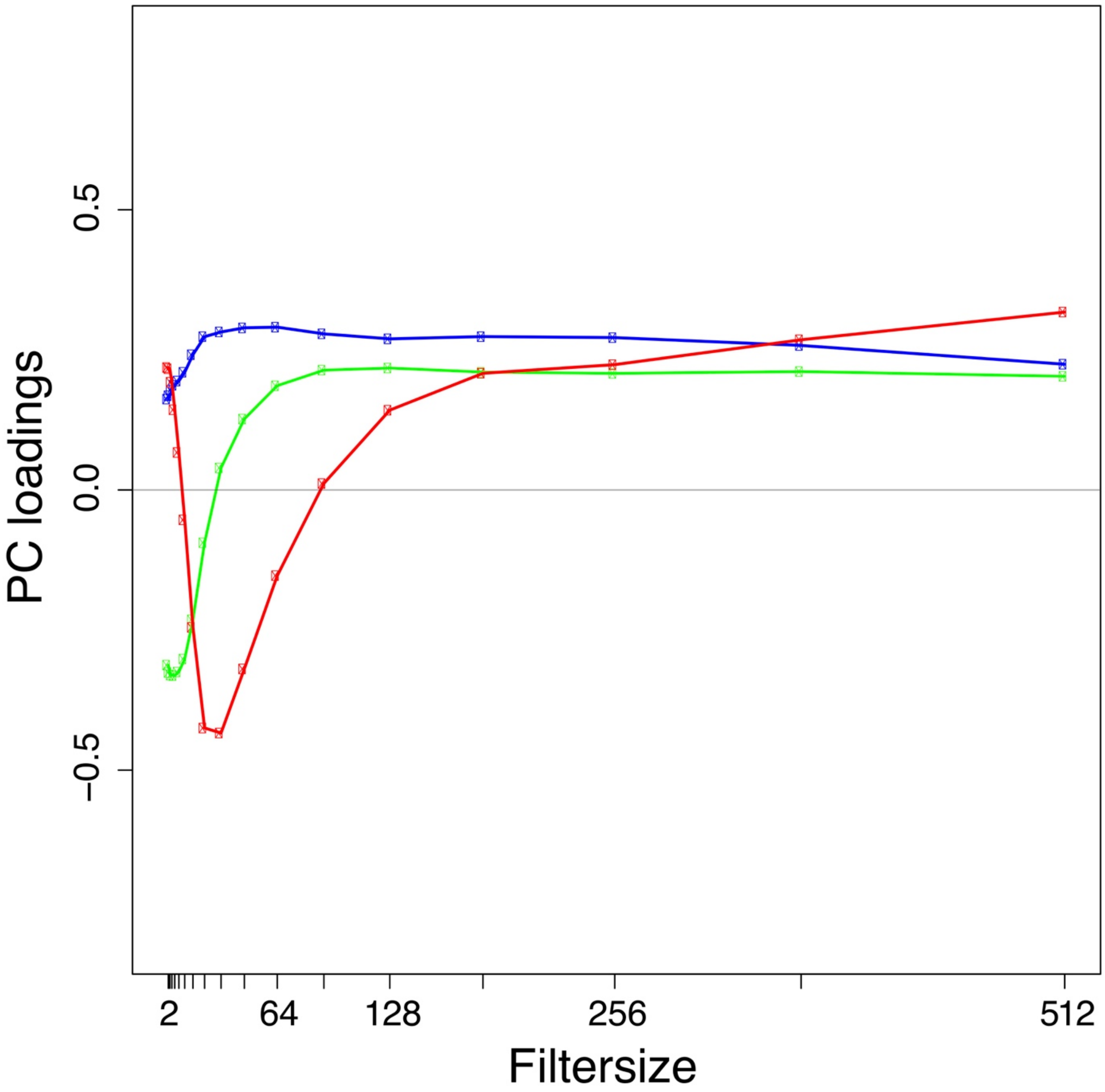

Supplementary Fig. 18 | Principal component loadings of belly pattern. Principal component loadings of pattern energy spectra across filter size for all species reveals how belly pattern elements of different sizes influence PC scores.
