# Supplementary material for: Ecological and geographical overlap drive plumage evolution and mimicry in woodpeckers
Source: Nat Commun. 2019 Apr 8;10:1602. doi: 10.1038/s41467-019-09721-w (PMC6453948; doi:10.1038/s41467-019-09721-w)
Supplement: Supplementary file 3 — Reporting Summary [file 41467_2019_9721_MOESM3_ESM.pdf]

nature research

Corresponding author(s): Eliot Miller

Reporting Summary

Nature Research wishes to improve the reproducibility of the work that we publish. This form provides structure for consistency and transparency in reporting. For further information on Nature Research policies, see Authors & Referees and the Editorial Policy Checklist.

Statistical parameters

When statistical analyses are reported, confirm that the following items are present in the relevant location (e.g. figure legend, table legend, main text, or Methods section).

n/a	Confirmed	
☐	☒	The exact sample size (*n*) for each experimental group/condition, given as a discrete number and unit of measurement
☐	☒	An indication of whether measurements were taken from distinct samples or whether the same sample was measured repeatedly
☐	☒	The statistical test(s) used AND whether they are one- or two-sided<br>*Only common tests should be described solely by name; describe more complex techniques in the Methods section.*
☐	☒	A description of all covariates tested
☐	☒	A description of any assumptions or corrections, such as tests of normality and adjustment for multiple comparisons
☐	☒	A full description of the statistics including central tendency (e.g. means) or other basic estimates (e.g. regression coefficient) AND variation (e.g. standard deviation) or associated estimates of uncertainty (e.g. confidence intervals)
☐	☒	For null hypothesis testing, the test statistic (e.g. *F*, *t*, *r*) with confidence intervals, effect sizes, degrees of freedom and *P* value noted<br>*Give P values as exact values whenever suitable.*
☒	☐	For Bayesian analysis, information on the choice of priors and Markov chain Monte Carlo settings
☒	☐	For hierarchical and complex designs, identification of the appropriate level for tests and full reporting of outcomes
☐	☒	Estimates of effect sizes (e.g. Cohen's *d*, Pearson's *r*), indicating how they were calculated
☐	☒	Clearly defined error bars<br>*State explicitly what error bars represent (e.g. SD, SE, CI)*

Our web collection on statistics for biologists may be useful.

Software and code

Policy information about availability of computer code

Data collection	ImageJ (v. 1.52) was used to collect the data for this project
Data analysis	R 3.4.2 was used for all analyses in this project. Within R, analyses employ standard R packages and a custom R package that we have extensively user-tested and made freely available on GitHub

For manuscripts utilizing custom algorithms or software that are central to the research but not yet described in published literature, software must be made available to editors/reviewers upon request. We strongly encourage code deposition in a community repository (e.g. GitHub). See the Nature Research guidelines for submitting code & software for further information.

Data

Policy information about availability of data

All manuscripts must include a data availability statement. This statement should provide the following information, where applicable:

- Accession codes, unique identifiers, or web links for publicly available datasets
- A list of figures that have associated raw data
- A description of any restrictions on data availability

All data is available in the manuscript or the supplementary materials.

Field-specific reporting

Please select the best fit for your research. If you are not sure, read the appropriate sections before making your selection.

☐ Life sciences ☐ Behavioural & social sciences ☒ Ecological, evolutionary & environmental sciences

For a reference copy of the document with all sections, see nature.com/authors/policies/ReportingSummary-flat.pdf

Ecological, evolutionary & environmental sciences study design

All studies must disclose on these points even when the disclosure is negative.

Study description	The study centerpiece was a multiple distance matrix regression, which sought to identify the power of four independent distance matrices to explain variation in a fifth dependent distance matrix. The four independent matrices were collated from established, publicly available sources: a phylogeny of the group (Shakya et al), geographic range overlaps (Sullivan et al), satellite data (NASA), climatic data (WorldClim).
Research sample	All available geographic records for all woodpeckers in the world were used.
Sampling strategy	One image was analyzed per species. Each image was taken to be the phenotypic average of that species.
Data collection	Eliot Miller and Russell Ligon collated all the data for the study.
Timing and spatial scale	All available site records were used in eBird, amounting to many millions of records of many years, with a preponderance of data in recent years.
Data exclusions	Some eBird records were excluded a priori based on best practices for citizen science/eBird data.
Reproducibility	The study is 100% reproducible, with all data freely available, and an entire R package already archived on GitHub that was written for, with examples, reproducing this work.
Randomization	NA
Blinding	NA

Did the study involve field work? ☐ Yes ☒ No

Reporting for specific materials, systems and methods

Materials & experimental systems

n/a	Involved in the study
☒	☐ Unique biological materials
☒	☐ Antibodies
☒	☐ Eukaryotic cell lines
☒	☐ Palaeontology
☒	☐ Animals and other organisms
☒	☐ Human research participants

Methods

n/a	Involved in the study
☒	☐ ChIP-seq
☒	☐ Flow cytometry
☒	☐ MRI-based neuroimaging
