# Supplementary material for: Ecological and geographical overlap drive plumage evolution and mimicry in woodpeckers
Source: Nat Commun. 2019 Apr 8;10:1602. doi: 10.1038/s41467-019-09721-w (PMC6453948; doi:10.1038/s41467-019-09721-w)
Supplement: Supplementary file 4 — Description of Additional Supplementary Files [file 41467_2019_9721_MOESM4_ESM.pdf]

Description of Additional Supplementary Files

File Name: Supplementary Data 1
Description: Species-level sample sizes of eBird point locations before and after spatial downsampling.
